# Clinical and laboratory features associated with macrophage activation syndrome in Still’s disease: data from the international AIDA Network Still’s Disease Registry

**DOI:** 10.1007/s11739-023-03408-3

**Published:** 2023-10-12

**Authors:** Paola Triggianese, Antonio Vitale, Giuseppe Lopalco, Henrique Ayres Mayrink Giardini, Francesco Ciccia, Ibrahim Al-Maghlouth, Piero Ruscitti, Petros Paul Sfikakis, Florenzo Iannone, Isabele Parente de Brito Antonelli, Martina Patrone, Kazi Nur Asfina, Ilenia Di Cola, Katerina Laskari, Carla Gaggiano, Abdurrahman Tufan, Paolo Sfriso, Lorenzo Dagna, Roberto Giacomelli, Andrea Hinojosa-Azaola, Gaafar Ragab, Lampros Fotis, Haner Direskeneli, Veronica Spedicato, Marilia Ambiel Dagostin, Daniela Iacono, Hebatallah Hamed Ali, Paola Cipriani, Jurgen Sota, Riza Can Kardas, Sara Bindoli, Corrado Campochiaro, Luca Navarini, Stefano Gentileschi, Eduardo Martín-Nares, Jiram Torres-Ruiz, Moustafa Ali Saad, Katerina Kourtesi, Fatma Alibaz-Oner, Gizem Sevik, Annamaria Iagnocco, Joanna Makowska, Marcello Govoni, Sara Monti, Maria Cristina Maggio, Francesco La Torre, Emanuela Del Giudice, José Hernández-Rodríguez, Elena Bartoloni, Giacomo Emmi, Maria Sole Chimenti, Armin Maier, Gabriele Simonini, Giovanni Conti, Alma Nunzia Olivieri, Maria Tarsia, Amato De Paulis, Alberto Lo Gullo, Ewa Więsik-Szewczyk, Ombretta Viapiana, Benson Ogunjimi, Samar Tharwat, Sukran Erten, Rossana Nuzzolese, Anastasios Karamanakos, Micol Frassi, Alessandro Conforti, Valeria Caggiano, Achille Marino, Gian Domenico Sebastiani, Antonio Gidaro, Enrico Tombetti, Francesco Carubbi, Giovanni Rubegni, Alessandra Cartocci, Alberto Balistreri, Claudia Fabiani, Bruno Frediani, Luca Cantarini

**Affiliations:** 1 Rheumatology, Allergology and Clinical Immunology, Department of Systems Medicine, University of Rome Tor Vergata, Rome, Italy; 2 University of Rome Tor Vergata, Rome, Italy; 3 Department of Medical Sciences, Surgery and Neurosciences, Research Center of Systemic Autoinflammatory Diseases, Behçet’s Disease Clinic and Rheumatology-Ophthalmology Collaborative Uveitis Center, University of Siena, Siena, Italy; 4 UOC Reumatologia, Azienda Ospedaliero-Universitaria Senese, ERN-RITA Center, Siena, Italy; 5 Rheumatology Unit, Department of Emergency and Organ Transplantation, University of Bari, Bari, Italy; 6 Rheumatology Division, Faculdade de Medicina, Hospital das Clínicas, Universidade de São Paulo, São Paulo, Brazil; 7 Department of Precision Medicine, Università Degli Studi Della Campania Luigi Vanvitelli, Naples, Italy; 8 Rheumatology Unit, Department of Medicine, College of Medicine, King Saud University, Riyadh, Saudi Arabia; 9 College of Medicine Research Center, College of Medicine, King Saud University, Riyadh, Saudi Arabia; 10 Rheumatology Unit, Department of Biotechnological and Applied Clinical Sciences, University of L’Aquila, L’Aquila, Italy; 11 Joint Academic Rheumatology Program, National and Kapodistrian University of Athens Medical School, Athens, Greece; 12 Division of Rheumatology, Department of Internal Medicine, Gazi University Faculty of Medicine, Ankara, Turkey; 13 Rheumatology Unit, Department of Medicine (DIMED), University of Padova, Padua, Italy; 14 Unit of Immunology, Rheumatology, Allergy and Rare Diseases, IRCCS San Raffaele Hospital, Milan, Italy; 15 Vita-Salute San Raffaele University, Milan, Italy; 16 Clinical and Research Section of Rheumatology and Clinical Immunology, Fondazione Policlinico Campus Bio-Medico, Via Álvaro del Portillo 200, 00128 Rome, Italy; 17 Rheumatology and Clinical Immunology, Department of Medicine, School of Medicine, University of Rome “Campus Biomedico”, Rome, Italy; 18 Department of Immunology and Rheumatology, Instituto Nacional de Ciencias Médicas y Nutrición Salvador Zubirán, Mexico City, Mexico; 19 Internal Medicine Department, Rheumatology and Clinical Immunology Unit, Faculty of Medicine, Cairo University, Giza, Egypt; 20 Faculty of Medicine, Newgiza University, 6th of October City, Egypt; 21 Third Department of Paediatrics, National and Kapodistrian University of Athens, General University Hospital “Attikon”, Athens, Greece; 22 Department of Internal Medicine, Division of Rheumatology, School of Medicine, Marmara University, Istanbul, Turkey; 23 Academic Rheumatology Center, Dipartimento Scienze Cliniche e Biologiche, Università degli Studi di Torino, Turin, Italy; 24 Department of Rheumatology, Medical University of Lodz, Lodz, Poland; 25 Rheumatology Unit, Department of Medical Sciences, Azienda Ospedaliero-Universitaria S. Anna-Ferrara, University of Ferrara, Ferrara, Italy; 26 Rheumatology Department, Istituto di Ricovero e Cura a Carattere Scientifico Policlinico S. Matteo Fondazione, University of Pavia, Pavia, Italy; 27 University Department Pro.Sa.M.I. “G. D’Alessandro”, University of Palermo, Palermo, Italy; 28 Clinical Pediatrics, University of Bari, Bari, Italy; 29 Pediatric and Neonatology Unit, Department of Maternal Infantile and Urological Sciences, Sapienza University of Rome, Polo Pontino, Latina, Italy; 30 Vasculitis Research Unit and Autoinflammatory Diseases Clinical Unit, Department of Autoimmune Diseases, Hospital Clinic of Barcelona, IDIBAPS, University of Barcelona, [European Reference Network (ERN) for Rare Immunodeficiency, Autoinflammatory and Autoimmune Diseases (RITA) Center], Barcelona, Spain; 31 Department of Medicine and Surgery, MED/16- Rheumatology, Università degli Studi di Perugia, P.Zza Università, 06123 Perugia, Italy; 32 Department of Experimental and Clinical Medicine, University of Florence, Florence, Italy; 33 Centre for Inflammatory Diseases, Department of Medicine, Monash Medical Centre, Monash University, Melbourne, Australia; 34 Rheumatology Unit, Department of Medicine, Central Hospital of Bolzano, Bolzano, Italy; 35 NEUROFARBA Department, Rheumatology Unit, Meyer Children’s University Hospital, University of Florence, Florence, Italy; 36 Pediatric Nephrology and Rheumatology Unit, AOU Policlinic G Martino, Messina, Italy; 37 Department of Woman, Child and of General and Specialized Surgery, University of Campania “Luigi Vanvitelli”, Naples, Italy; 38 Section of Clinical Immunology, Department of Translational Medical Sciences, University of Naples Federico II, Naples, Italy; 39 Department of Translational Medical Sciences, Center for Basic and Clinical Immunology Research (CISI), World Allergy Organisation Center of Excellence, University of Naples Federico II, Naples, Italy; 40 Unit of Rheumatology, Department of Medicine, ARNAS Garibaldi Hospital, Catania, Italy; 41 Department of Internal Medicine, Pulmonology, Allergy and Clinical Immunology, Central Clinical Hospital of the Ministry of National Defence, Military Institute of Medicine, National Research Institute, Warsaw, Poland; 42 Rheumatology Unit, Department of Medicine, University and Azienda Ospedaliera Universitaria Integrata of Verona, Verona, Italy; 43 Antwerp Unit for Data Analysis and Computation in Immunology and Sequencing, University of Antwerp, Antwerp, Belgium; 44 Antwerp Center for Translational Immunology and Virology, Vaccine and Infectious Disease Institute, University of Antwerp, Antwerp, Belgium; 45 Department of Paediatrics, Antwerp University Hospital, Antwerp, Belgium; 46 Center for Health Economics Research and Modeling Infectious Diseases, Vaccine and Infectious Disease Institute, University of Antwerp, Antwerp, Belgium; 47 Rheumatology and Immunology Unit, Internal Medicine Department, Mansoura University, Mansoura, Egypt; 48 Department of Internal Medicine, Faculty of Medicine, Horus University, New Damietta, Egypt; 49 Department of Rheumatology, Faculty of Medicine Ankara City Hospital, Ankara Yıldırım Beyazıt University, Ankara, Turkey; 50 Joint Academic Rheumatology Program, First Department of Propaedeutic and Internal Medicine, National and Kapodistrian University of Athens, Mikras Asias Street 75 Goudi, 11527 Athens, Greece; 51 Rheumatology and Clinical Immunology, Spedali Civili and Department of Clinical and Experimental Sciences, University of Brescia, [European Reference Network (ERN) for Rare Immunodeficiency, Autoinflammatory and Autoimmune Diseases (RITA) Center], Brescia, Italy; 52 U.O. Medicina Generale, Ospedale San Paolo di Civitavecchia, ASL Roma 4, Civitavecchia, Rome, Italy; 53 Unit of Pediatric Rheumatology, ASST Gaetano Pini-CTO, Milan, Italy; 54 UOC di Reumatologia, Azienda Ospedaliera San Camillo Forlanini, Rome, Italy; 55 Department of Biomedical and Clinical Sciences Luigi Sacco, Luigi Sacco Hospital, University of Milan, Milan, Italy; 56 Internal Medicine, Fatebenefratelli Hospital, Milan, Italy; 57 Department of Biomedical and Clinical Sciences, University of Milan, Milan, Italy; 58 Department of Life, Health & Environmental Sciences and Internal Medicine and Nephrology Unit, Department of Medicine, University of L’Aquila and ASL Avezzano-Sulmona-L’Aquila, San Salvatore Hospital, L’Aquila, Italy; 59 Ophthalmology Unit, Department of Medicine, Surgery and Neurosciences, University of Siena and Azienda Ospedaliero-Universitaria Senese [European Reference Network (ERN) for Rare Immunodeficiency, Autoinflammatory and Autoimmune Diseases (RITA) Center], Siena, Italy; 60 Bioengineering and Biomedical Data Science Lab, Department of Medical Biotechnologies, University of Siena, Siena, Italy; 61 Policlinico “Le Scotte”, Viale Bracci 1, 53100 Siena, Italy

**Keywords:** Arthritis, Autoinflammatory diseases, Diagnosis, MAS, Prognosis

## Abstract

To characterize clinical and laboratory signs of patients with Still’s disease experiencing macrophage activation syndrome (MAS) and identify factors associated with MAS development. Patients with Still’s disease classified according to internationally accepted criteria were enrolled in the AutoInflammatory Disease Alliance (AIDA) Still’s Disease Registry. Clinical and laboratory features observed during the inflammatory attack complicated by MAS were included in univariate and multivariate logistic regression analysis to identify factors associated to MAS development. A total of 414 patients with Still’s disease were included; 39 (9.4%) of them developed MAS during clinical history. At univariate analyses, the following variables were significantly associated with MAS: classification of arthritis based on the number of joints involved (*p* = 0.003), liver involvement (*p* = 0.04), hepatomegaly (*p* = 0.02), hepatic failure (*p* = 0.01), axillary lymphadenopathy (*p* = 0.04), pneumonia (*p* = 0.03), acute respiratory distress syndrome (*p* < 0.001), platelet abnormalities (*p* < 0.001), high serum ferritin levels (*p* = 0.009), abnormal liver function tests (*p* = 0.009), hypoalbuminemia (*p* = 0.002), increased LDH (*p* = 0.001), and LDH serum levels (*p* < 0.001). At multivariate analysis, hepatomegaly (OR 8.7, 95% CI 1.9–52.6, *p* = 0.007) and monoarthritis (OR 15.8, 95% CI 2.9–97.1, *p* = 0.001), were directly associated with MAS, while the decade of life at Still’s disease onset (OR 0.6, 95% CI 0.4–0.9, *p* = 0.045), a normal platelet count (OR 0.1, 95% CI 0.01–0.8, *p* = 0.034) or thrombocytosis (OR 0.01, 95% CI 0.0–0.2, *p* = 0.008) resulted to be protective. Clinical and laboratory factors associated with MAS development have been identified in a large cohort of patients based on real-life data.

**Supplementary Information:**

The online version contains supplementary material available at 10.1007/s11739-023-03408-3.

## Introduction

Still’s disease is a rare systemic autoinflammatory polygenic disorder capable of affecting both adults (adult-onset Still’s disease, AOSD) and pediatric patients (systemic juvenile idiopathic arthritis, sJIA) [1, 2]. Noteworthy, increasing evidence suggests that AOSD and sJIA represent a pathological *continuum* rather than different clinical entities [3]. Still’s disease recognizes a wide spectrum of non-specific symptoms and clinical manifestations including fever, arthralgia (with or without arthritis), skin rash (especially salmon-pink rash), pharyngodynia, lymphadenopathy, hepato-splenomegaly, and serositis [4, 5]. Neurological, renal, and ophthalmological involvement can rarely occur, thus worsening clinical outcome and complicating the treatment approach [6]. Common laboratory findings include neutrophilic leukocytosis, elevated C-reactive protein (CRP), increased liver enzymes, and high ferritin levels with low glycosylated ferritin (≤ 20%) [7]. Clinical disease course includes three significant patterns with a different distribution and prognosis, consisting of a self-limited or monophasic pattern, a polycyclic intermittent disease, and a chronic-articular course [8, 9]. However, more recently, a dichotomous pattern with a predominant systemic disease and a predominant articular course has been proposed, with the former characterized by higher body temperature, ferritin serum levels, liver enzymes and thrombocytosis and the latter characterized by a more severe articular involvement [10–12].

The diagnosis of AOSD is essentially clinical and the accurate exclusion of mimickers such as malignancies, infections, and other autoimmune diseases is necessary to confirm the diagnosis [13]. Classification criteria may be useful in recognizing Still’s disease, with Yamaguchi’s criteria and Fautrel’s criteria being the most frequently employed in adults and the International League of Associations for Rheumatology (ILAR) criteria and/or the Pediatric Rheumatology INternational Trials Organization (PRINTO) criteria used in the pediatric setting [14–17].

Hyperactivation of monocytes/macrophages might play central role in the development of macrophage activation syndrome (MAS), one of the most severe and life-threating complications of Still’s disease [8, 9]. This complication has been described in up to 15% of patients with this disease; the mortality rate ranges between 2.3 and 16% in such cases [18]. MAS is a secondary hemophagocytic lymphohistiocytosis (HLH) associated with autoimmune diseases [19]. As described by different authors, HLH and MAS are not discrete diseases, but they are the *continuum* of hemophagocytic disorders sharing common pathways of impaired cytotoxicity leading to a “cytokine inflammatory storm” [1, 20, 21].

The classification criteria more frequently used for MAS in Still’s disease have been validated for patients with sJIA [22]. However, they are commonly used also for other systemic autoinflammatory diseases. According to this set of criteria, MAS classification may occur when a febrile patient with known or suspected Still’s disease shows high serum ferritin (> 684 ng/ml) and at least two items among decreased platelet count (< 181 × 10^9^/l), decreased fibrinogen serum levels (< 360 mg/dl), increased aspartate aminotransferase (AST, > 48 U/l), and increased triglycerides (> 156 mg/dl) [22]. A second score, defined HScore, has been developed and validated for the diagnosis of reactive HLH, both in rheumatologic and non-rheumatologic conditions [23]. It is more frequently used in adult patients. A further set of criteria developed on behalf of the Histiocytic Society (HLH-2004) is also used, but it is primarily intended for genetic forms of HLH [24].

MAS can occur either at the time of diagnosis or later during patients’ clinical history, but specific defined predictive factors are lacking or require to be confirmed on large population studies [19]. Similarly, clinical and laboratory clues capable of simplifying identification of MAS would also be needed to improve patients’ outcome. On this basis, the aim of the present study is to characterize clinical and laboratory signs of patients with Still’s disease experiencing MAS and to identify factors associated with MAS development.

## Methods

This cohort study was based on data collected in the International AutoInflammatory Disease Alliance (AIDA) Registry dedicated to Still’s disease [25]. Patients with Still’s disease, classified according to internationally accepted criteria (Yamaguchi and/or Fautrel and/or ILAR and/or PRINTO criteria [14–17]) were enrolled between 2021 June and 2023 January.

The developing of MAS was defined according to the fulfilment of 2016 MAS criteria and/or the HLH-2004 criteria and/or on the basis of an HScore > 250 (MAS probability > 90%); patients never developing MAS accounted for the comparison group [22–24].

Demographic data included sex, ethnicity, age at the Still’s disease onset, diagnostic delay, and Still’s disease duration. Disease course was defined as monocyclic, polycyclic, and chronic-articular course [26]; the disease course remained unknown in patients in whom the course was already undefined at the last follow-up. Patients were also stratified in two subgroups based on the age at the Still’s disease onset: patients with age at onset < 16 years old were classified as pediatric.

Laboratory variables considered in the analysis were: erythrocyte sedimentation rate (ESR), CRP, aspartate aminotransferase (AST), alanine aminotransferase (ALT), β2-microglobulin, lactate dehydrogenase (LDH), direct and total bilirubin, gamma-glutamyltransferase (GGT), alkaline phosphatase (AP). These variables were recorded either during the attack leading to MAS development or at the worst episode of Still’s disease (based on the occurrence or non-occurrence of MAS); they were considered increased in accordance with each laboratory reference limit.

Hypoalbuminemia was defined as < 3.5 g/dl; hypergammaglobulinemia (polyclonal gammopathy) was identified as a broad-based peak or band in the gamma region on serum protein electrophoresis. Anemia was defined as Hb ≤ 12 g/dl; platelets count ≥ 450,000/mm^3^ defined thrombocytosis in adults while ≥ 500,000/mm^3^ defined thrombocytosis in childhood [27]; platelets count ≤ 150,000/mm^3^ defined thrombocytopenia [28]. Abnormal liver function was defined as the occurrence of increased levels of AST and/or ALT and/or direct/total bilirubin and/or GGT, and/or AP, while liver involvement consisted of having abnormal liver function and/or hepatomegaly (identified by ultrasound and/or radiological documentations). Referring to the classification criteria proposed by Yamaguchi et al. [14], hyperferritinemia was defined as a serum ferritin level higher than 3000 ng/ml and leukocytosis as a white blood count exceeding 15,000/mm^3^.

Diagnosis of pleuritis and/or pneumonia, pericarditis, and peritonitis was based on ultrasound and/or radiological documentations; similarly, lymphadenopathy and splenomegaly were confirmed by ultrasound and/or computed tomography scans.

The occurrence of the following clinical variables referring to the attack leading to MAS development was also registered: highest body temperature (°C), skin rash, thoracic and/or abdominal pain, arthralgia, myalgia, conjunctivitis, kidney involvement, orchitis, neurologic involvement, and severe complications including fulminant hepatic failure and acute respiratory distress syndrome (ARDS). Arthritis was classified as monoarticular (1 joint), oligoarticular (2–4 joints), and polyarticular (≥ 5 joints. Still’s disease severity was assessed according to the systemic scores proposed by Pouchot et al. [29] and Rau et al. [30].

### Statistical analysis

Continuous variables were described as mean and standard deviation (SD) or as median and interquartile range (IQR), where appropriate. The Kolmogorov–Smirnov test was used to determine normality. Categorical variables were reported as frequencies and percentages. Differences in variables were compared using the unpaired *t* test for normally distributed quantitative data, and the Mann–Whitney test for non-normally distributed quantitative data. The Chi-squared test or the Fisher’s exact test were used to analyze association between categorical variables. Univariate and multivariate logistic regression analyses were used to identify factors associated with MAS. Univariate logistic regression analyses were performed to assess the unadjusted association between MAS and clinical/laboratory parameters while stepwise multivariate logistic regression analyses were used to identify independent factors associated with MAS. The variables entering the multivariate model were statistically significant and clinically relevant at univariate analyses (*p* < 0.05). Only the patients characterized by a good retrospective data collection (80% of data correctly inserted into the AIDA registry) were included in the univariate regression analysis and in the subsequent multivariate model. Supplementary table 1 provides demographic, clinical and laboratory data accounting for independent variables at univariate and multivariate logistic regression; the MAS development (yes/no) represented the dependent variable. The level of significance was 0.05. All statistical analyses were conducted using STATA 17/MP2 (StataCorp. 2021. Stata Statistical Software: Release 17. College Station, TX: StataCorp LLC).

## Results

We included 414 patients. Ethnicity was as follows: caucasic (*n* = 344, 83.1%), Arab (*n* = 39, 9.4%), Hispanic (*n* = 18, 4.3%), Africans (*n* = 7, 1.7%), indigenous peoples of South America (*n* = 3, 0.7%), Asian (*n* = 2, 0.5%), indigenous peoples of North America (*n* = 1, 0.24%). Women represented 59.9% of the study population (*n* = 248) (Table 1).

**Table 1:** Demographic data from the study population and disease course

	Study population, *N* = 414	MAS, *N* = 39	Non-MAS, *N* = 375	*p*
Age at Still’s disease onset, yrs, Mean ± SD	32.54 ± 17.14	25.26 ± 16.49	33.25 ± 16.75	0.003
Diagnostic delay, yrs Median (IQ; range)	0.2 (0.8; 0–22.9)	0.2 (0.9; 0–21.9)	0.1 (0.2; 0–20.5)	0.101
Still’s disease duration, yrs Median (IQ; range)	0.2 (0.8; 0–22)	0.1 (0.2; 0–20.4)	0.2 (0.9; 0–22)	0.07
Females (*N*/%)	248/59.9	25/64.1	223/59.5	0.61
Pediatric age onset (*N*/%)	63/15.2	11/28.2	52/13.9	0.02
Monocyclic course (*N*/%)	129/31.2	15/38.5	114/30.4	0.3
Polycyclic course (*N*/%)	104/25.1	13/33.3	91/24.3	0.22
Systemic course (*N*/%)	233/56.3	28/71.8	205/54.7	0.04
Chronic-articular course (*N*/%)	86/20.8	3/7.7	83/22.1	0.04
Unknown course (*N*/%)	95/22.9	8/20.5	87/23.2	0.7

*p* values refer to comparisons between MAS and non-MAS groups; they were obtained with Mann–Whitney *U* test for quantitative data and Chi-square test for qualitative data

*IQ* interquartile range, *MAS* macrophage activation syndrome, *SD* standard deviation, *yrs* years

In the whole cohort, the median disease duration at enrollment was 0.2 year (IQR 0.8, range 0–22). The mean age at the Still’s disease onset was 32.54 ± 17.14 years (range 0.8–79.7) with a median diagnostic delay of 0.2 years (IQR 0.8, range 0–22.9) and a female to male (F:M) ratio of 1.47. Patients had mainly a monocyclic disease course (*n* = 129, 31.2%) followed by a polycyclic course (*n* = 104, 25.1%) and then chronic-articular courses (*n* = 86, 20.8%). However, the disease course remained still unknown in 22.9% of the cohort (*n* = 95) owing to the short follow up period.

Patients with age at the Still’s disease onset < 16 years old represented 15.2% of the study population (*n* = 63, mean age at diagnosis 10.25 ± 2 years). A similar sex distribution occurred in pediatric patients (F:M ratio 1.1), while patients with age at the disease onset ≥ 16 years old (*n* = 353, mean age at diagnosis 38.17 ± 15 yrs) showed a slightly higher female prevalence (F:M ratio 1.55).

Both in pediatric and adult patients, comparable prevalence occurred for the monocyclic (28.6% *vs* 31.6%, respectively, *p* = 0.63) and polycyclic (27% vs 24.2%, respectively, *p* = 0.53) disease course; conversely, the chronic-articular type was significantly less frequent in the pediatric context (30.2% in adults *vs* 19.1% among children, *p* = 0.046). An unknown Still’s disease course was registered in 14.3% of pediatric-onset cases and 24.2% of adult-onset cases, *p* = 0.08).

### MAS patients

Patients with MAS represented 9.4% (*n* = 39) of the whole cohort, with a F:M ratio of 1.8 (Table 1). Thirty-six (92.3%) patients fulfilled 2016 criteria for MAS [22]; 3 patients fulfilled the HLH-2004 criteria and/or the HScore criteria [23, 24]. Thirty-six (92.3%) patients developed MAS at the start of Still’s disease; the remaining 3 (7.7%) patients developed MAS during a subsequent Still’s disease flare. Most of MAS patients were Caucasics (*n* = 35, 89.7%); 2 Arabs (5.1%), 1 (2.5%) Hispanic, and 1 (2.5%) indigenous from North America were also observed. The age at Still’s disease onset was 25.26 ± 16.49 years, with a median diagnostic delay of 0.2 yr (IQR 0.9, range 0–21.9). None of them died.

Patients with age at the disease onset < 16 y.o. represented 28.2% of the MAS group, with a F:M ratio of 1.5, and a median age at the disease onset of 10.9 years (IQR 9, range 0.8–16 years); patients with age at the disease onset ≥ 16 y.o. showed a F:M ratio of 1.8, and a median age at the disease onset of 30.65 years (IQR 22.1, range 17.3–75.5 years).

### Non-MAS patients

Patients with non-MAS were the largest part of the cohort (*n* = 375, 90.6%) with a F:M ratio of 1.5 (Table 1). Caucasic ethnicity included 82.4% of this subgroup (*n* = 309). The age at the disease onset was 33.25 ± 16.75 years, with a median diagnostic delay of 0.1 year (IQR 0.2, range 0–20.5).

Patients with age at the disease onset < 16 y.o. represented 13.9% of the group with a F:M ratio of 0.96, and a median age at the disease onset of 9.9 years (IQR 8.1, range 0.8–15.9 years); patients with age at the disease onset ≥ 16 y.o. showed a F:M ratio of 1.6, and a median age at the disease onset of 34.9 years (IQR 20.8, range 16.1–79.7 years).

### Comparing MAS and non-MAS patients

#### Clinical features

The Still’s disease onset at age < 16 y.o. resulted significantly more frequent in the MAS group compared with the non-MAS group (*p* = 0.02). Accordingly, the age at onset resulted lower in MAS than in non-MAS patients (*p* = 0.003) (Table 1).

A chronic-articular disease course was significantly more prevalent in non-MAS group compared with the MAS group (*p* = 0.04, Table 1). However, in MAS group, a slightly higher prevalence occurred for a monocyclic disease course while the polycyclic course was registered in a similar percentage between MAS and non-MAS groups (Table 1).

At univariate analyses, performed on patients with complete data insertion (39 patients with MAS and 330 patients with no MAS history), the following clinical manifestations were significantly associated with MAS: liver involvement (*p* = 0.04), hepatomegaly (*p* = 0.02), hepatic failure (*p* = 0.01), and the development of axillary lymphadenopathy (*p* = 0.04). In addition, the development of pneumonia (*p* = 0.03) and ARDS (*p* < 0.001) were significantly associated with MAS. Similarly, the identification of scleritis during the inflammatory attach was found significantly associated with MAS development (*p* = 0.003). The mean value of the highest body temperature reached during attacks was higher in MAS than in non-MAS patients (*p* = 0.03).

Abdominal pain showed a trend toward a higher frequency in MAS than in non-MAS patients without a statistically significant difference (see Table 2).

**Table 2:** Clinical findings from the study groups

Clinical features	MAS, *N* = 39	Non-MAS, *N* = 375	*p* value
Skin rash, *N* (%)	25 (64.1)	230 (61.3)	0.74
Splenomegaly, *N* (%)	11 (28.2)	126 (33.6)	0.49
Liver involvement, *N* (%)	19 (48.7)	123 (32.8)	0.047
Hepatomegaly, *N* (%)	16 (41)	86 (22.9)	0.02
Lymphadenopathy, *N* (%)	25 (64.1)	182 (48.5)	0.065
Pneumonia, *N* (%)	4 (10.3)	17 (4.5)	0.12
Serositis (any site), *N* (%)	13 (33.3)	102 (27.2)	0.42
Pleuritis, *N* (%)	8 (20.5)	50 (13.3)	0.22
Pericarditis, *N* (%)	4 (10.3)	50 (13.3)	0.59
Peritonitis, *N* (%)	1 (2.6)	3 (0.8)	0.28
Thoracic pain, *N* (%)	4 (10.3)	37 (9.9)	0.94
Abdominal pain, *N* (%)	10 (25.6)	46 (12.3)	0.06
Arthritis, *N* (%)	21 (53.8)	216 (57.6)	0.65
Myalgia, *N* (%)	23 (59)	186 (49.6)	0.27
Eye involvement, *N* (%)	5 (12.8)	15 (4)	0.02
Scleritis, *N* (%)	3 (7.7)	0 (0)	< 0.001
Conjunctivitis, *N* (%)	2 (5.1)	11 (3)	0.46
Kidney, *N* (%)	2 (5.1)	7 (1.9)	0.18
Orchitis, *N* (%)	0/0 (0)	3 (0.8)	0.58
Neurologic, *N* (%)	2 (5.1)	9 (2.4)	0.31
ARDS, *N* (%)	4 (10.3)	1 (0.3)	< 0.001
Fulminant hepatic failure, *N* (%)	2 (5.1)	1 (0.3)	0.001
Highest BT, °C; median (IQR)	40 (0.65)	39.5 (1.0)	0.02
Highest BT			
≥ 39 °C, *N* (%)	38 (97.4)	317 (84.5)	0.03

*p* values were obtained with Student’s *t* test for quantitative data and Chi-square test or Fisher exact test (according to the sample size and expected frequencies) for qualitative data

*ARDS* acute respiratory distress syndrome, *BT* body temperature, *IQR* interquartile range, *MAS* macrophage activation syndrome

**Fig. 1:**
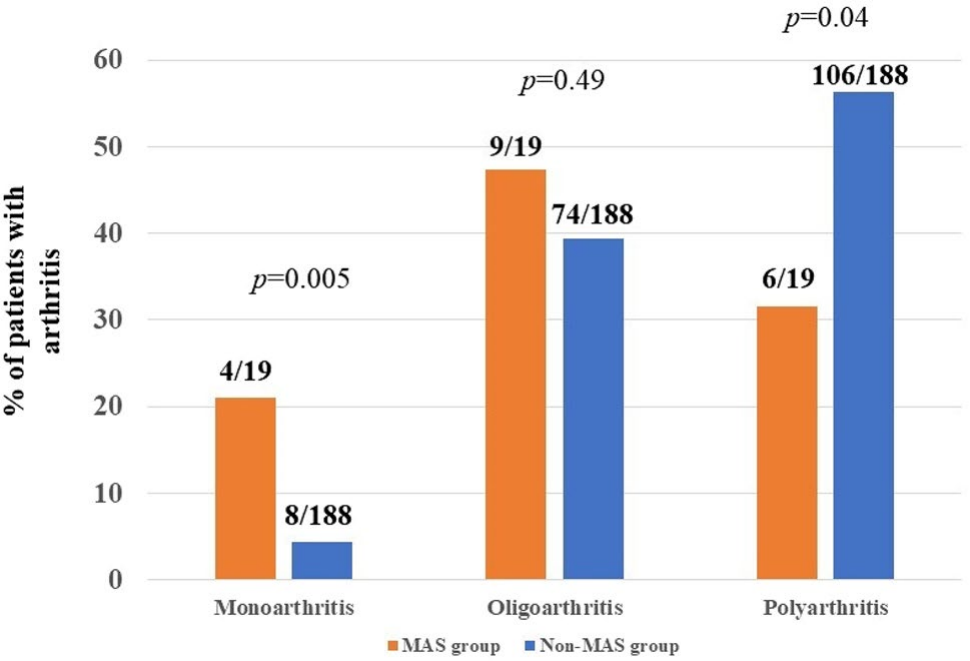
Types of arthritis in Still’s patients according to the number of joints involved. Patients with macrophage activation syndrome (MAS) showed significantly different distributions in the number of joints involved by arthritis, when compared to patients without MAS. *p* values were obtained with the Chi-squared test or the Fisher’s exact test were according to frequency counts and expected frequencies

The classification of arthritis based on the number of joints involved (monoarthritis/oligoarthritis/polyarthritis) was significantly associated with MAS (*p* = 0.003). In particular, considering all patients with arthritis, the monoarticular subtype was more present in the MAS group than in non-MAS group (4/19, 21% vs 8/188, 4.3%, *p* = 0.005); 2 out of the 4 patients with MAS and monoarthritis were pediatric patients. Joints involved in these cases were the knee (*n* = 3) and the wrist (n = 1). Polyarthritis was more prevalent in non-MAS than in MAS (106/188, 56.4% vs 6/19, 31.6%, *p* = 0.04). The oligoarthritic subtype recognized a similar distribution between the two groups (9/19, 47.4% in the MAS group vs 74/188, 39.4% in the non-MAS group, *p* = 0.49) (Fig. 1).

Although the occurrence of serositis (pleuritis and/or pericarditis, and/or peritonitis) showed a similar prevalence in MAS and non-MAS subjects, pleuritis resulted twofold more frequent than pericarditis in MAS patients; conversely, pleuritis and pericarditis showed a comparable occurrence in non-MAS patients (see Table 2).

The prevalence of other clinical Still’s disease related manifestation was similar in the two study groups as described in Table 2.

#### Laboratory findings

At univariate analysis, the following laboratory parameter results were significantly associated with MAS: platelet abnormalities (*p* < 0.001), high serum ferritin levels (*p* = 0.003), abnormal liver function tests (*p* = 0.009)—especially AST (*p* = 0.002), ALT (*p* = 0.04), total bilirubin (*p* = 0.049), direct bilirubin (*p* = 0.004)—and hypoalbuminemia (*p* = 0.002) (Table 3). Moreover, patients with increased LDH were prevalent in MAS group than in non-MAS (*p* = 0.001), while LDH serum levels were higher among MAS patients than in non-MAS patients (*p* < 0.001) (Table 3).

**Table 3:** Laboratory findings from the study groups

Laboratory findings	MAS, *N* = 39	Non-MAS, *N* = 375	*p* value
ESR, mm/h; mean ± SD	75.5 ± 29.4	79.5 ± 32.4	0.43
CRP mg/dl; median (IQR)	16 (20.5)	13.35 (23.175)	0.21
Anemia, *N* (%)	23 (58.9)	204 (54.4)	0.69
Leukocytosis, *N* (%)	29 (74.4)	255 (68)	0.58
White blood cell count, cells/mmc; median (IQR)	18,000 (4150)	16,955 (5400)	0.15
Absolute neutrophil count, cells/mmc; median (IQR)	15,662 (5320)	14,434.5 (5782)	0.06
Thrombocytosis, *N* (%)	3 (9.4)	83 (22.1)	0.04
Normal platelet count, *N* (%)	20 (51.3)	270 (72)	0.03
Thrombocytopenia, *N* (%)	16 (41)	22 (5.9)	< 0.001
Abnormal liver function, *N* (%)	26 (66.7)	153 (40.8)	0.009
Increased AST, *N* (%)	25 (64.1)	119 (31.7)	0.002
Increased ALT, *N* (%)	22 (56.4)	120 (32)	0.04
Conjugated hyperbilirubinemia, *N* (%)	5 (12.8)	6 (1.6)	0.004
Total hyperbilirubinemia, *N* (%)	4 (10.3)	6 (1.6)	0.049
Hypoalbuminemia, *N* (%)	21 (53.8)	90 (24)	0.002
Increased ferritin, *N* (%)	36 (92.3)	318 (84.8)	0.18
Ferritin, ng/ml; median (IQR)	6908 (30,106.5)	1559.5 (5355)	0.003
Increased LDH, *N* (%)	29 (74.4)	146 (38.9)	0.001
LDH (U/L, mean ± SD)	790 (1906.5)	487 (219)	< 0.001
Hypergammaglobulinemia, *N* (%)	8 (20.5)	63 (16.8)	0.99
Increased β2-microglobulin, *N* (%)	5 (12.8)	80 (21.3)	0.65

*p* values were obtained with Student’s *t* test or Mann–Whitney *U* test (according to data distribution) for quantitative data and Chi-square test for qualitative data

*ALT* alanine aminotransferase, *AST* aspartate aminotransferase, *CRP* C-reactive protein, *ESR* erythrocyte sedimentation rate, *IQR* interquartile range, *LDH* lactate dehydrogenase, *MAS* macrophage activation syndrome, *SD* standard deviation

Levels of ESR and CRP were not found to discriminate MAS and non-MAS groups in a significant fashion; similarly, anemia, leukocytosis, the white blood cells count, and the absolute neutrophil count were comparable between the two groups (Table 3).

#### Multivariate analysis

**Fig. 2:**
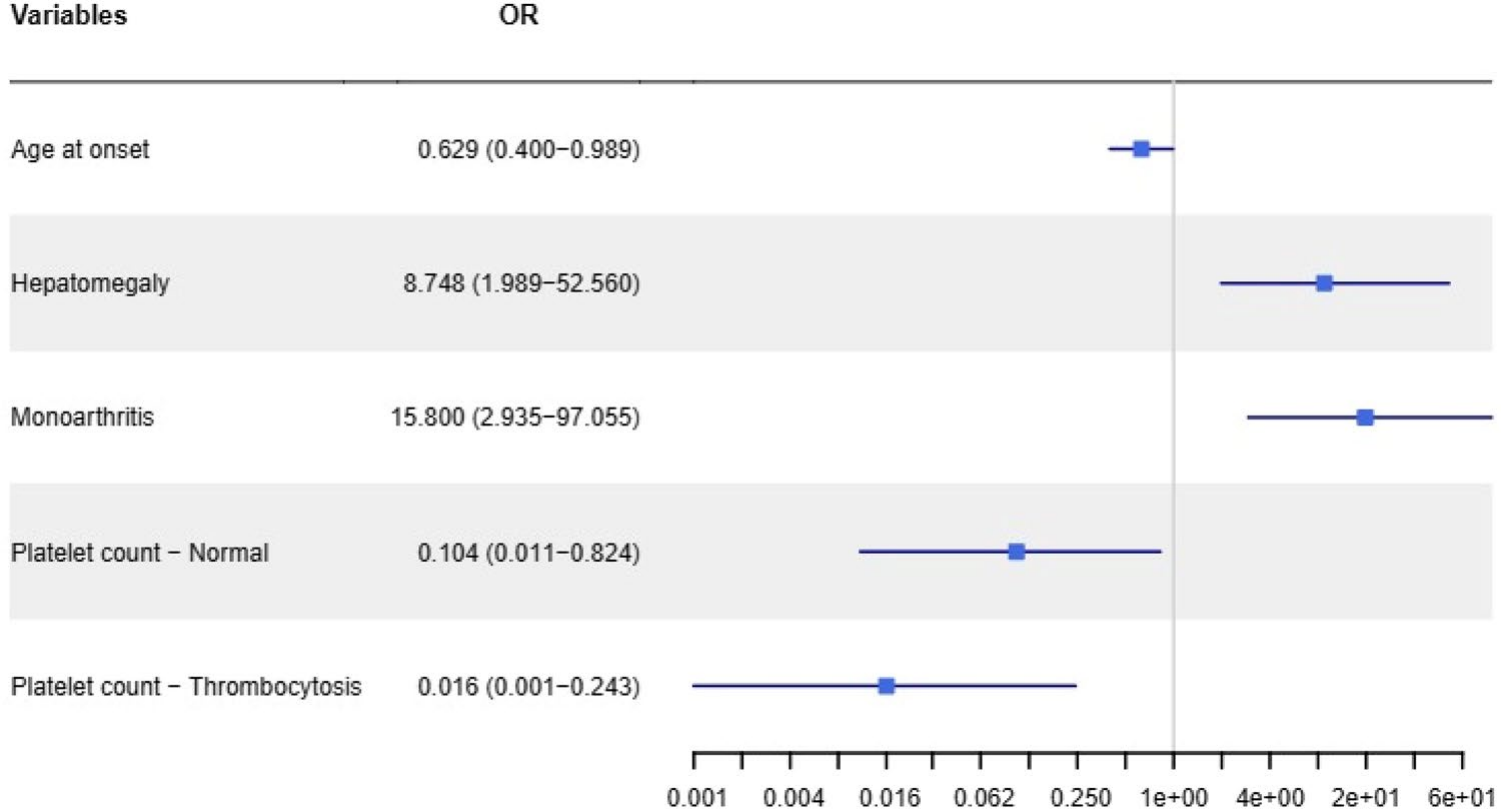
Results of multivariate logistic regression analysis. Odds Ratios and 95% confidence interval of variables significantly associated with MAS development; *x*-axis was represented with logarithmic scale to facilitate graphical description. As noted, patients with a normal platelet count or even more with thrombocytosis have a lower probability to develop MAS; similarly, this probability decreases along with the increase of the age at the time of disease onset, provided as decades of life at the time of disease onset. On the contrary, patients with monoarthritis and hepatomegaly are more prone to develop MAS. The figure represents the outcome of a stepwise multivariate logistic regression analysis. Forty non-MAS patients characterized by an inadequate retrospective data collection (less than 80% of data required) were not considered for regression analysis

After the stepwise procedure, the variables that resulted statistically significant were hepatomegaly (OR 8.7, 95% CI 1.9–52.6, *p* = 0.007) and monoarthritis (OR: 15.8, 95% CI 2.9–97.1, *p* = 0.001), that were directly associated with MAS development. The age at Still’s disease onset (OR 0.6, 95% CI 0.4–0.9, *p* = 0.045, the OR indicates the risk for a 10-year increment of age), a normal platelet count (OR 0.1, 95% CI 0.01–0.8, *p* = 0.034) and the presence of thrombocytosis (OR 0.02, 95% CI 0.0–0.2, *p* = 0.008) were inversely associated with MAS development (Fig. 2).

#### Clinical severity scores

**Fig. 3:**
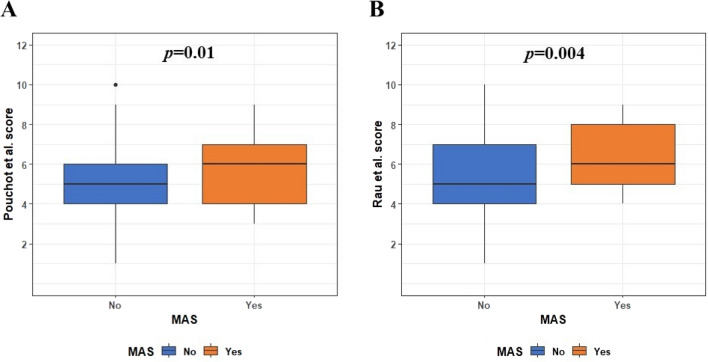
Systemic scores for Still’s disease severity. Patients with macrophage activation syndrome (MAS) reported significantly higher Pouchot et al. score [29] and Rau et al. score [30], compared to the non-MAS group, as represented in **A** and **B**, respectively. *p* values were obtained using the Mann–Whitney test for non-normally distributed continuous data. The lower whiskers correspond to 1.5 times the first quartile; the upper whiskers correspond to 1.5 times the third quartile

The median value of the systemic score for Still’s disease severity was 6 (IQR = 3) in the MAS group and 6 (IQR = 2) in the non-MAS group according to Pouchot et al.; it was significantly higher in the MAS group (*p* = 0.01). The same result occurred by analyzing clinical manifestations using the systemic score modified by Rau et al., whose median value resulted to be 6 (3) in the MAS group and 5 (3) in the non-MAS group; it was significantly higher in the former group (*p* = 0.004) (Fig. 3). A systemic Pouchot score ≥ 7 was significantly more frequent among MAS patients than in non-MAS patients (12/39 versus 60/357 patients, *p* = 0.02); conversely, the systemic score by Rau et al. was not significantly different among groups (13/39 versus 82/357, *p* = 0.11).

## Discussion

The lack of clinical and reliable clues to early and effective detection of MAS often concur to the inadequate treatment and to the poor outcome for this disorder [31, 32]. We suggest here defined clinical indicators capable of associating with MAS development in Still’s disease and useful at improving the early detection of this life-threatening complication. In particular, the number of joints involved by arthritis, the occurrence of liver involvement, pneumonia, ARDS, and axillary lymphadenopathy appear associated with MAS development, along with the identification of thrombocytopenia, hyperferritinemia, hypoalbuminemia, increased LDH, and abnormal liver function tests.

In particular, the presence of hepatomegaly and monoarthritis have shown to be associated with the development of MAS. The association with monoarthritis confirms the higher frequency of MAS among patients with the systemic course rather than among subjects with the chronic-articular disease, which is typically characterized by a more prominent articular involvement.

Patients with a normal platelet count or even more with thrombocytosis have a lower probability to develop MAS, as observed at multivariate analysis. Similarly, an older age at disease onset results protective for MAS, with a reduction of the risk as the patients’ age increases at the start of symptoms. To further support this finding, a significantly higher prevalence of pediatric patients results in the MAS group compared with the non-MAS group, while age at onset was lower in MAS than in non-MAS patients. These results are clinically relevant, as the age at Still’s disease onset may occur at any age and the risk for MAS development reduces as the decades of life increase at the onset of Still’s disease.

The lower prevalence of the chronic-articular disease course in MAS patients reflects the well-known features of the systemic type, which is characterized by a more pronounced cytokine storm-induced inflammation [33]. Consequently, our findings confirm that MAS arises more frequently in patients with a more prominent inflammatory phenotype*.* Polyarthritis was more prevalent in Still’s disease patients who do not develop MAS. This is in accordance with the lower frequency of MAS among patients with the chronic-articular disease course and the lower inflammatory burden observed in this type of disease [33, 34]. This also corroborates the protective role of increasing age against MAS development, as the chronic-articular Still’s disease course was significantly more frequent among patients with adult disease onset.

At univariate analyses, the present study defines liver involvement and the occurrence of axillary lymphadenopathy as clinical manifestations significantly associated with MAS development. These clues agree with findings observed in previous studies [35, 36]. In particular, strong associations have already been found between liver involvement and ferritin serum levels, which may act as a pathogenic protein contributing to the development of a self-perpetuating cytokine storm [37, 38]. Therefore, patients with liver involvement might indicate exaggerated inflammatory responses in Still’s disease patients [39]. Previous studies also described that Still’s disease patients with MAS are more likely to have hepatomegaly than Still’s disease without MAS and that patients with liver involvement have an almost sixfold higher risk of MAS [35, 40]. Noteworthy, MAS as HLH can present with wide range of hepatic dysfunction ranging from mild abnormalities of transaminases to liver failure. Particularly, MAS patients with liver failure might experience ARDS and/or respiratory failure due to pneumonia [41, 42]. In this regard, lung involvement has been recognized as an emergent cause of mortality and a marker of poor prognosis in Still’s disease [43]; also, it has been found associated with MAS and seems to define a distinct clinical and immunologic pattern in such cases [44]. The present work corroborates all these observations.

Pleuritis and pericarditis represent relevant features for Still’s disease [45]. Our data show that pleuritis is twofold frequent than pericarditis in Still’s disease patients developing MAS. This is in agreement with previously reported evidence supporting that Still’s disease patients with pleuritis also experience a higher frequency of disseminated intravascular coagulation and MAS [46]. Furthermore, patients with Still’s disease and serosal involvement have proved to be more likely to develop MAS [36, 47].

Concerning laboratory investigations, our results document that thrombocytopenia, elevated serum ferritin, LDH levels, abnormal liver function—in particular AST, ALT, total bilirubin, direct bilirubin, and hypoalbuminemia—are significantly associated with MAS. Moreover, patients with increased LDH are more prevalent in the MAS group than in non-MAS group. These findings support previous data about the role of laboratory investigations in identifying patients with MAS. In particular, dynamic changes in some laboratory data, especially platelet count, were found to differentiate MAS from flare-ups of autoimmune diseases, while changes in liver enzymes levels, high ferritin levels and white blood cells may enhance an early diagnosis of MAS [48, 49]. Accordingly, a recent study aimed at identifying valuable serum laboratory markers reflecting MAS disease activity documented that significant changes in platelet count, LDH levels, and D-dimers accounted for the most valuable indicators of MAS in patients with systemic JIA [50]. Other authors documented that extremely elevated serum LDH levels represented useful diagnostic markers for MAS along with moderate to severe lymphopenia [51]. Similarly, the levels of AST, LDH, and platelet count resulted to be associated with a poor prognosis in patients with rheumatic diseases and MAS, including those with Still’s disease [52].

Both the systemic Pouchot score [29] and the modified systemic score by Rau et al*.* [30], used to assess disease severity, have proved to be significantly higher in the MAS group compared with non-MAS patients. In this regard, the systemic score values by Pouchot et al*.* had already been found to be significantly higher in patients developing MAS [35]. The present study confirms the usefulness of the systemic score by Pouchot et al*.* as clinical tool to be used in patients with suspected MAS. In addition, our results indicate that the systemic score modified by Rau et al*.* may have a similar role in warning physicians to the possible development of MAS. Noteworthy, a Pouchot score higher than 7 was significantly more frequent in the MAS group than in non-MAS patients, confirming the capability of this cut-off in recognizing patients at higher risk of death [53].

Limitations of the study include the retrospective design and the relatively small number of patients developing MAS. Diagnostic criteria for MAS in Still’s disease used in this study have not been validated for adult patients with Still’s disease [22]. Nevertheless, they are generally used for patients with autoinflammatory diseases and suspected MAS. Also, data from pediatric patients were meshed with those referring to adult patients. In this regard, the age at disease onset appears to be a variable associated with MAS development rather than a confounding factor, and this was strongly highlighted by combining pediatric-onset patients with adults.

Of note, the lack of deaths could be related to a better management and an earliest recognition of MAS in recent times compared with what recorded in previous decades [18]; however, it could also conceal a selection bias, as investigators may have omitted the recruitment of deceased patients. If so, the power of factors identified in this study in recognizing the most severe MAS patients could be partially affected and future studies should evaluate whether the results achieved in this study are superimposed on those that will be obtained from patients with fatal MAS. However, based on real-life data reflecting the daily clinical practice, this study largely confirms the findings previously obtained from smaller cohorts of patients. It thus provides the clinical and laboratory clues to take into account when dealing with Still’s disease patients suspected to complicate with MAS.

In conclusion, the risk of developing MAS is higher among pediatric patients. Subjects with systemic disease course, monoarthritis, liver involvement, pneumonia, ARDS and axillary lymphadenopathy are more likely to develop MAS; this probability is lower among patients with polyarthritis and chronic-articular disease course. Thrombocytopenia, abnormalities in liver function tests, serum ferritin levels, LDH, and hypoalbuminemia represent laboratory findings to be primarily considered for early detection of patients developing MAS.

### Supplementary Information

Below is the link to the electronic supplementary material.

Supplementary file1 (DOCX 208 KB)

## Data Availability

The datasets generated during and/or analyzed during the current study are available from the corresponding author on reasonable request.
