# Correction: Clinical and laboratory features associated with macrophage activation syndrome in Still’s disease: data from the international AIDA Network Still’s Disease Registry

**DOI:** 10.1007/s11739-023-03511-5

**Published:** 2023-12-27

**Authors:** Paola Triggianese, Antonio Vitale, Giuseppe Lopalco, Henrique Ayres Mayrink Giardini, Francesco Ciccia, Ibrahim Al-Maghlouth, Piero Ruscitti, Petros Paul Sfikakis, Florenzo Iannone, Isabele Parente de Brito Antonelli, Martina Patrone, Kazi Nur Asfina, Ilenia Di Cola, Katerina Laskari, Carla Gaggiano, Abdurrahman Tufan, Paolo Sfriso, Lorenzo Dagna, Roberto Giacomelli, Andrea Hinojosa-Azaola, Gaafar Ragab, Lampros Fotis, Haner Direskeneli, Veronica Spedicato, Marilia Ambiel Dagostin, Daniela Iacono, Hebatallah Hamed Ali, Paola Cipriani, Jurgen Sota, Riza Can Kardas, Sara Bindoli, Corrado Campochiaro, Luca Navarini, Stefano Gentileschi, Eduardo Martín-Nares, Jiram Torres-Ruiz, Moustafa Ali Saad, Katerina Kourtesi, Fatma Alibaz-Oner, Gizem Sevik, Annamaria Iagnocco, Joanna Makowska, Marcello Govoni, Sara Monti, Maria Cristina Maggio, Francesco La Torre, Emanuela Del Giudice, José Hernández-Rodríguez, Elena Bartoloni, Giacomo Emmi, Maria Sole Chimenti, Armin Maier, Gabriele Simonini, Giovanni Conti, Alma Nunzia Olivieri, Maria Tarsia, Amato De Paulis, Alberto Lo Gullo, Ewa Więsik-Szewczyk, Ombretta Viapiana, Benson Ogunjimi, Samar Tharwat, Sukran Erten, Rossana Nuzzolese, Anastasios Karamanakos, Micol Frassi, Alessandro Conforti, Valeria Caggiano, Achille Marino, Gian Domenico Sebastiani, Antonio Gidaro, Enrico Tombetti, Francesco Carubbi, Giovanni Rubegni, Alessandra Cartocci, Alberto Balistreri, Claudia Fabiani, Bruno Frediani, Luca Cantarini

**Affiliations:** 1 Rheumatology, Allergology and Clinical Immunology, Department of Systems Medicine, University of Rome Tor Vergata, Rome, Italy; 2 University of Rome Tor Vergata, Rome, Italy; 3 Department of Medical Sciences, Surgery and Neurosciences, Research Center of Systemic Autoinflammatory Diseases, Behçet’s Disease Clinic and Rheumatology-Ophthalmology Collaborative Uveitis Center, University of Siena, Siena, Italy; 4 UOC Reumatologia, Azienda Ospedaliero-Universitaria Senese, ERN-RITA Center, Siena, Italy; 5 Rheumatology Unit, Department of Emergency and Organ Transplantation, University of Bari, Bari, Italy; 6 Rheumatology Division, Faculdade de Medicina, Hospital das Clínicas, Universidade de São Paulo, São Paulo, Brazil; 7 Department of Precision Medicine, Università Degli Studi Della Campania Luigi Vanvitelli, Naples, Italy; 8 Rheumatology Unit, Department of Medicine, College of Medicine, King Saud University, Riyadh, Saudi Arabia; 9 College of Medicine Research Center, College of Medicine, King Saud University, Riyadh, Saudi Arabia; 10 Rheumatology Unit, Department of Biotechnological and Applied Clinical Sciences, University of L’Aquila, L’Aquila, Italy; 11 Joint Academic Rheumatology Program, National and Kapodistrian University of Athens Medical School, Athens, Greece; 12 Division of Rheumatology, Department of Internal Medicine, Gazi University Faculty of Medicine, Ankara, Turkey; 13 Rheumatology Unit, Department of Medicine (DIMED), University of Padova, Padua, Italy; 14 Unit of Immunology, Rheumatology, Allergy and Rare Diseases, IRCCS San Raffaele Hospital, Milan, Italy; 15 Vita-Salute San Raffaele University, Milan, Italy; 16 Clinical and Research Section of Rheumatology and Clinical Immunology, Fondazione Policlinico Campus Bio-Medico, Via Álvaro del Portillo 200, 00128 Rome, Italy; 17 Rheumatology and Clinical Immunology, Department of Medicine, School of Medicine, University of Rome “Campus Biomedico”, Rome, Italy; 18 Department of Immunology and Rheumatology, Instituto Nacional de Ciencias Médicas y Nutrición Salvador Zubirán, Mexico City, Mexico; 19 Internal Medicine Department, Rheumatology and Clinical Immunology Unit, Faculty of Medicine, Cairo University, Giza, Egypt; 20 Faculty of Medicine, Newgiza University, 6th of October City, Egypt; 21 Third Department of Paediatrics, National and Kapodistrian University of Athens, General University Hospital “Attikon”, Athens, Greece; 22 Department of Internal Medicine, Division of Rheumatology, School of Medicine, Marmara University, Istanbul, Turkey; 23 Academic Rheumatology Center, Dipartimento Scienze Cliniche e Biologiche, Università degli Studi di Torino, Turin, Italy; 24 Department of Rheumatology, Medical University of Lodz, Lodz, Poland; 25 Rheumatology Unit, Department of Medical Sciences, Azienda Ospedaliero-Universitaria S. Anna-Ferrara, University of Ferrara, Ferrara, Italy; 26 Rheumatology Department, Istituto di Ricovero e Cura a Carattere Scientifico Policlinico S. Matteo Fondazione, University of Pavia, Pavia, Italy; 27 University Department Pro.Sa.M.I. “G. D’Alessandro”, University of Palermo, Palermo, Italy; 28 Clinical Pediatrics, University of Bari, Bari, Italy; 29 Pediatric and Neonatology Unit, Department of Maternal Infantile and Urological Sciences, Sapienza University of Rome, Polo Pontino, Latina, Italy; 30 Vasculitis Research Unit and Autoinflammatory Diseases Clinical Unit, Department of Autoimmune Diseases, Hospital Clinic of Barcelona, IDIBAPS, University of Barcelona, [European Reference Network (ERN) for Rare Immunodeficiency, Autoinflammatory and Autoimmune Diseases (RITA) Center], Barcelona, Spain; 31 Department of Medicine and Surgery, MED/16- Rheumatology, Università degli Studi di Perugia, P.Zza Università, 06123 Perugia, Italy; 32 Department of Experimental and Clinical Medicine, University of Florence, Florence, Italy; 33 Centre for Inflammatory Diseases, Department of Medicine, Monash Medical Centre, Monash University, Melbourne, Australia; 34 Rheumatology Unit, Department of Medicine, Central Hospital of Bolzano, Bolzano, Italy; 35 NEUROFARBA Department, Rheumatology Unit, Meyer Children’s University Hospital, University of Florence, Florence, Italy; 36 Pediatric Nephrology and Rheumatology Unit, AOU Policlinic G Martino, Messina, Italy; 37 Department of Woman, Child and of General and Specialized Surgery, University of Campania “Luigi Vanvitelli”, Naples, Italy; 38 Section of Clinical Immunology, Department of Translational Medical Sciences, University of Naples Federico II, Naples, Italy; 39 Department of Translational Medical Sciences, Center for Basic and Clinical Immunology Research (CISI), World Allergy Organisation Center of Excellence, University of Naples Federico II, Naples, Italy; 40 Unit of Rheumatology, Department of Medicine, ARNAS Garibaldi Hospital, Catania, Italy; 41 Department of Internal Medicine, Pulmonology, Allergy and Clinical Immunology, Central Clinical Hospital of the Ministry of National Defence, Military Institute of Medicine, National Research Institute, Warsaw, Poland; 42 Rheumatology Unit, Department of Medicine, University and Azienda Ospedaliera Universitaria Integrata of Verona, Verona, Italy; 43 Antwerp Unit for Data Analysis and Computation in Immunology and Sequencing, University of Antwerp, Antwerp, Belgium; 44 Antwerp Center for Translational Immunology and Virology, Vaccine and Infectious Disease Institute, University of Antwerp, Antwerp, Belgium; 45 Department of Paediatrics, Antwerp University Hospital, Antwerp, Belgium; 46 Center for Health Economics Research and Modeling Infectious Diseases, Vaccine and Infectious Disease Institute, University of Antwerp, Antwerp, Belgium; 47 Rheumatology and Immunology Unit, Internal Medicine Department, Mansoura University, Mansoura, Egypt; 48 Department of Internal Medicine, Faculty of Medicine, Horus University, New Damietta, Egypt; 49 Department of Rheumatology, Faculty of Medicine Ankara City Hospital, Ankara Yıldırım Beyazıt University, Ankara, Turkey; 50 Joint Academic Rheumatology Program, First Department of Propaedeutic and Internal Medicine, National and Kapodistrian University of Athens, Mikras Asias Street 75 Goudi, 11527 Athens, Greece; 51 Rheumatology and Clinical Immunology, Spedali Civili and Department of Clinical and Experimental Sciences, University of Brescia, [European Reference Network (ERN) for Rare Immunodeficiency, Autoinflammatory and Autoimmune Diseases (RITA) Center], Brescia, Italy; 52 U.O. Medicina Generale, Ospedale San Paolo di Civitavecchia, ASL Roma 4, Civitavecchia, Rome, Italy; 53 Unit of Pediatric Rheumatology, ASST Gaetano Pini-CTO, Milan, Italy; 54 UOC di Reumatologia, Azienda Ospedaliera San Camillo Forlanini, Rome, Italy; 55 Department of Biomedical and Clinical Sciences Luigi Sacco, Luigi Sacco Hospital, University of Milan, Milan, Italy; 56 Internal Medicine, Fatebenefratelli Hospital, Milan, Italy; 57 Department of Biomedical and Clinical Sciences, University of Milan, Milan, Italy; 58 Department of Life, Health & Environmental Sciences and Internal Medicine and Nephrology Unit, Department of Medicine, University of L’Aquila and ASL Avezzano-Sulmona-L’Aquila, San Salvatore Hospital, L’Aquila, Italy; 59 Ophthalmology Unit, Department of Medicine, Surgery and Neurosciences, University of Siena and Azienda Ospedaliero-Universitaria Senese [European Reference Network (ERN) for Rare Immunodeficiency, Autoinflammatory and Autoimmune Diseases (RITA) Center], Siena, Italy; 60 Bioengineering and Biomedical Data Science Lab, Department of Medical Biotechnologies, University of Siena, Siena, Italy; 61 Policlinico “Le Scotte”, Viale Bracci 1, 53100 Siena, Italy

**Correction: Internal and Emergency Medicine (2023) 18:2231–2243** 10.1007/s11739-023-03408-3

In the original version of this article, the given and family names of Alberto Lo Gullo were incorrectly structured. The name was displayed correctly in all versions publication.

The original article has been corrected.

