# Supplementary material for: Clinical and laboratory features associated with macrophage activation syndrome in Still’s disease: data from the international AIDA Network Still’s Disease Registry
Source: Intern Emerg Med. 2023 Oct 12;18(8):2231–43. doi: 10.1007/s11739-023-03408-3 (PMC10635948; doi:10.1007/s11739-023-03408-3)
Supplement: Supplementary file 1 — Supplementary file1 (DOCX 208 KB) [file 11739_2023_3408_MOESM1_ESM.docx]

**Supplementary Table 1**: list of demographic, clinical and laboratory manifestations used as independent variables at univariate logistic regression analysis, with the development of macrophage activation syndrome (MAS) being the dependent variable. The variables in bold were significantly associated with MAS development at univariate analysis (p<0.05) and were included as independent variables in the multivariate model. Abbreviations: alanine aminotransferase (ALT); aspartate aminotransferase (AST).

| Demographic variables: sex, **age at onset**, **age at diagnosis**, disease duration at diagnosis. |
| --- |
| Disease course (**chronic-articular**, monocyclic, polycyclic, still unknown) |
| Number of acute inflammatory attacks/year |
| Features of fever: mean duration of the fever episodes, **highest body temperature reached during attacks (°C)**, fever course (continuous, remittent, transient), pattern of fever during the day (no patterns, quotidian, twice quotidian) |
| Presence (Yes/No) of the following clinical manifestations observed either during the attack with MAS or during the worst episode of Still’s disease: pharyngodynia, salmon-coloured evanescent skin rash, other skin manifestations, splenomegaly, lymphadenopathy, sites of lymphadenopathy (laterocervical, **axillary**, inguinal, mediastinal/thoracic, mesenterial/abdominal), **liver involvement**, **hepatomegaly**, acute hepatitis, fulminant hepatis, **hepatic failure**, thoracic pain not associated to serositis, **pneumonia**, pleuritis, pericarditis, abdominal pain, peritonitis, abdominal effusions, vomiting, diarrhea, myalgia, arthralgia, arthritis, **classification of arthritis based on the number of joints involved** (**monoarthritis**, oligoarthritis, **polyarthritis**), **inflammatory ocular involvement** (uveitis, **scleritis**, conjunctivitis), kidney involvement, orchitis, neurological involvement, disseminated intravascular coagulation, thrombotic thrombocytopenic purpura, diffuse alveolar hemorrhage, **acute respiratory distress syndrome**, cardiac complications, vascular complications |
| Presence (Yes/No) of the following laboratory condition recorded at the attack with MAS or during the worst episode of Still’s disease: anemia, leukocytosis, **normal platelet count**, **thrombocytopenia**, **thrombocytosis**, increased serum ferritin levels, **abnormal liver function tests**, **abnormal AST**, **abnormal ALT**, abnormal gamma-glutamyl transferase, abnormal alkaline phosphatase, **total bilirubin**, **conjugated bilirubin**, unconjugated bilirubin, hypergammaglobulinemia, increased immunoglobulins A, increased immunoglobulins M, increased immunoglobulins G, **hypoalbuminemia**, increased beta-2 microglobulin, **increased lactic dehydrogenase** |
| The following laboratory parameter referring to the attack with MAS or during the worst episode of Still’s disease: erythrocyte sedimentation rate (mm/1h), C reactive protein (mg/dl), the higher white blood cell count (cells/mm^3^), the higher percentage (%) of neutrophils, **serum ferritin level (ng/ml)**, serum beta-2 microglobulin value (mg/L), **lactic dehydrogenase serum value (U/L)** |
| Systemic severity score: **according to Pouchot et al** [29], **according to Rau et al** [30] |
